# On Gastrotomy

**Published:** 1825-03

**Authors:** C. Waller


### ART. VI.—*On Gastrotomy.* By C. WALLER, Esq.

EVERY one who deviates from the common beaten track, and proposes any thing new towards the improvement of any art, science, or profession, must make up his mind to be assailed in every way that envy on the one hand, or ignorance on the other, can suggest. Thus it was with the illustrious Hunter, many of whose doctrines were at first treated with contempt; but, "magna est veritas, et prevalebit," we find, now the grave has closed over him, that the most eminent men of the day are trying to outvie each other in their eulogies of him.

The operation of opening the abdomen for the removal of enlarged ovaria and other diseases, has been for some years recommended by Dr. Blundell, in his obstetric lectures. The experiments which he instituted on animals, with a view of throwing light on this subject, are detailed with great precision in his little work just published, entitled "Physiological and Pathological Researches;" and I think they fully justify us in believing that wounds of the peritoneum are not necessarily, nor perhaps generally, attended with those fatal effects which most of us have been taught to believe. That gastrotomy is attended with danger, no one will deny, and we must expect that fatal consequences will sometimes ensue: but I would ask, which of the established operations of surgery has not occasionally destroyed life, especially on its first introduction? A few failures, therefore, of this operation will not warrant us in condemning the practice altogether.

A very interesting paper on this subject has been published by Mr. Lizars, in the 81st Number of the Edinburgh Medical and Surgical Journal, wherein several cases are related, in which gastrotomy, to the extent of many inches, was performed, without destroying, or even obviously endangering life; and in two patients enlarged ovaria were extracted. In the present limited state of our experience, I conceive that any single fact tending to elucidate this point of practice is of service, as it is only by the accumulation of single facts that we can be enabled to decide the important question, whether recoveries from extensive gastrotomy are to be looked upon as exceptions, or as belonging to a general rule? I shall, therefore, make no further apology for relating the effects it produced on a female I lately had an opportunity of seeing.

The patient herself gave me the account; it therefore cannot be expected that I could obtain all the particulars of her case; but, as my object is merely to add one more to the list of recoveries after exposure of the abdominal cavity, I do not consider the tedious detail of every day's symptoms at all necessary. Suffice it to say, that a wound, to the extent of from three to four inches, was made into the cavity of the peritoneum, through the linea alba, beginning a little below the umbilicus. Towards the close of the operation, the patient became in a great degree insensible, though but little blood was lost; re-action followed in a few hours, and afterwards a diffused sore-ness, arising, I presume, from general inflammation. This was subdued by the usual means. The patient was extremely nervous, delicate, and irritable, and had a teazing cough: this latter circumstance, probably, prevented its healing by adhesion, for a period of twelve weeks elapsed before union was com-pleted, during which time the discharge was copious. She is now as well as she was previously to the operation. The scar on the abdomen, of course, remains conspicuous.

In one of the cases above alluded to, and where Mr. Lizars himself operated, he says, after describing the incision, "I now proceeded to examine the state of the tumor, but, to my asto-nishment, could find none." I have reason to believe the same thing occurred to the female whose case I have related; and, if so, it strongly points out the necessity of carefully ascertaining the existence of a tumor previous to an operation; and, even when this is determined on, would it not be prudent at first to make a small opening, just sufficient to admit the finger, in order to guard against the possibility of being deceived. The abdo-men of many hysterical patients is so large and so hard, that it may easily be mistaken for an enlarged ovarium, on a superficial examination. I well recollect a case of this kind, which occur-red to myself: the patient said she was seven months' gone with child, and she had milk secreted in both breasts. She com-plained of so much pain about the cervix uteri, that she con-sented to submit to an examination; when I found the uterus of its natural unimpregnated size, and consequently considered it to be an enlarged ovarium. I requested my late valued friend, Mr. Crampton, to visit this case with me, when, to my great astonishment, the tumor was gone.

In Mr. Lizars' case, the wound, or at least nearly the whole of it, healed by adhesion,—in the case related above, by granula-tion; it seems, therefore, that in either case patients may recover.

In Mr. L.'s case, there was no collapse after the operation; this symptom may, therefore, be regarded as accidental, and not as a necessary concomitant. In both, there appears to have been diffused inflammation of the peritoneum; but, in the case I have related, it occurred while the abdominal cavity was ex-posed to the air, as the healing process had not commenced, and yet the patient survived. Her constitution also was radi-cally bad for any serious operation.

In Mr. L.'s case, the incision was commenced two inches from the ensiform cartilage, and extended to the crista of the pubis; the wound was, therefore, in this case, of a much greater extent.

One precaution taken by Mr. L. was, I think, very judicious, and which I shall therefore transcribe:—"As inflammation," he observes, "appears to be generally induced by exposure to cold, and as these cases succeeded so well in America, I desired the room to be heated to 80° Fahrenheit." When the temperature of the room had arrived at this heat, the operation was commenced. After the incision was made, the intestines, of course, protruded, and they were enveloped in a towel dipped in water about 98°." This is a hint which is worth taking advantage of in future operations of this nature; and I do confidently hope the time is not distant when schirrus and other diseases of the ovary will be radically and safely (as far as that term will apply to any operation of surgery,) cured by extraction.

*Aldersgate-street; January,* 1825.

